# Vulnerability of female dentists to workplace violence

**DOI:** 10.1038/s41598-025-10953-8

**Published:** 2025-07-09

**Authors:** Yasmin Fonseca, Mayara Rangel, Nikolaos Angelakopoulos, Irina Makeeva, João Marcos da Costa Ribeiro, Luiz Renato Paranhos, Ademir Franco

**Affiliations:** 1 Division of Forensic Dentistry, Faculdade São Leopoldo Mandic, Campinas, SP Brazil; 2 Department of Dentistry, Universidade Cruzeiro do Sul, São Paulo, SP Brazil; 3 Department of Orthodontics and Dentofacial Orthopedics, University of Bern, Freiburgstrasse 7, Bern, 3010 Switzerland; 4 Department of Therapeutic Stomatology, Institute of Dentistry, Sechenov University, Moscow, Russia; 5 Dentistry post-graduate researcher, Universidade Federal de Uberlândia, Uberlândia, MG Brazil; 6 Department of Orthodontics, School of Dentistry, Universidade Federal de Uberlândia, Uberlândia, MG Brazil

**Keywords:** Dentist, Female, Forensic dentistry, Violence, Women, Dentistry, Forensic dentistry

## Abstract

Workplace violence is a significant public health issue, particularly affecting female healthcare professionals. This cross-sectional study aimed to assess the vulnerability of female dentists in São Paulo, Brazil, to workplace violence. A sample of 165 female dentists participated by responding to a structured digital questionnaire. The findings revealed that approximately 55% of the respondents had experienced some form of violence, with verbal and psychological abuse being the most common. Intimidation and stalking by patients or their companions were significant predictors of such incidents, increasing the likelihood of exposure by 16-fold (*p* < 0.001) and 3-fold (*p* = 0.001), respectively. Dentists in public healthcare services and those assisted by clinical staff were more frequently exposed to workplace violence. Among the respondents, 28% sought help, with psychological treatment being the most utilized form of support (*p* < 0.001), especially among less experienced professionals (*p* = 0.034). Although certain environmental and professional variables, such as working alone or during night shifts, did not reach statistical significance, they remained important factors for risk assessment. The results highlight the intimate nature of dental practice as a potential vulnerability factor, particularly due to the prolonged close contact with patients and their companions. This study underscores the importance of implementing preventive and protective strategies, including digital monitoring tools and institutional support, and advocates for accessible mental health resources to help mitigate the impact of workplace violence on female dentists.

**Supplementary Information:**

The online version contains supplementary material available at 10.1038/s41598-025-10953-8.

## Introduction

Violence against women persists as a public health issue^1^ despite research efforts and the development of healthcare strategies and protective policies^2^. Violence can be physical, psychological, or sexual^3^, and can impact individuals at home and in their workplace^4^. The latter is defined as any form of violence experienced during occupational activities^4^. Alarming rates of workplace violence are observed among healthcare workers, especially females^5^. This phenomenon may be related to the predominance of women in healthcare professions, as well as their lower hierarchical status^5^ and less favorable working conditions compared to their male counterparts. Brazil, for example, has 427,048 registered dentists, with approximately 30% located in the state of São Paulo. It is estimated that 64% are women. When dental technicians and assistants are included, the proportion of women in Dentistry rises to 72% (https://crosp.org.br/*).*

Workplace violence against female dentists can be perpetrated by patients, patients’ companions, and clinical and non-clinical staff members^6^. They can occur early in college (against female dental students) or later in the clinical practice. Dentists usually work privately and in a small space with restricted access^7^ – hence the vulnerability. In addition, clinical interactions normally occur in what is called “intimate space” in the field of proxemics^8^ (up to 0.4 m between individuals)^9,10^. Furthermore, treatments in specialties such as orthodontics, prosthodontics, and periodontology often last longer than in other medical fields^11,12^, increasing female dentists’ vulnerability to violence due to prolonged exposure to patients and their companions.

The most common types of violence experienced by these professionals include verbal and physical aggression^6^as well as bullying^13^. Studies have reported varying prevalence rates of violence, depending on the population studied and the time period examined^6,13–15^. Although violence can never be justified, previous healthcare research suggested that it may be associated with patients’ dissatisfaction with services and supplies^16^. One of the main consequences of workplace violence against women is mental health trauma. This type of trauma can impair their performance and increase the risk of workplace absenteeism, leading to financial and social issues, such as damage to reputation and isolation. A common objective of studies in the field is to investigate workplace violence in order to reduce the risks of exposure to harm^17^. However, progress in this area has been slow.

The present study aimed to (I) assess the current situation of violence perpetrated by patients and their companions against female dentists in the state of São Paulo, Brazil, (II) understand their workplace conditions, and (III) propose strategies to promote a healthier work environment for women in Dentistry.

## Materials and methods

### Ethical aspects and study design

This was an observational, analytical, cross-sectional study that followed the ethical aspects of the Declaration of Helsinki revised in 2024. Ethical approval was obtained from the institutional Committee of Ethics in Human Research (protocol: 74294223.4.0000.5374; approval number: 6.591.436) at Faculdade São Leopoldo Mandic, Campinas, São Paulo, Brazil.

This study was reported in accordance with the guidelines of the Enhancing the Quality and Transparency of Health Research (EQUATOR) network, specifically following the Strengthening the Reporting of Observational Studies in Epidemiology (STROBE) statement^18^.

### Sample and participants

Participants were selected based on eligibility criteria that included only Brazilian female dentists over 18 years of age, working in public or private services in the state of São Paulo, Brazil. The exclusion criteria consisted of dentists who were (i) not registered with the Regional Dental Council of São Paulo, (ii) inactive during the study period (e.g., due to public holidays, annual or maternity leave, or other absences), (iii) unwilling to participate, (iv) legally or administratively prohibited from practicing, or (v) worked only part-time in São Paulo (defined as spending less than 50% of their professional workload in the state).

The sample size was estimated based on the total number of dentists in the state of São Paulo at the time the study (*n* = 111,893), including the total number of female dentists (*n* = 78,325). Next, a recent systematic literature review² was considered to estimate the frequency of violence against oral healthcare providers (from 15 to 54%), and the expected response rate of the participants (between 53% and 92.59%). The data obtained helped to calculate a minimum sample size of 163 participants for the present study, assuming a population proportion of 54%, margin of error of 5%, and confidence level of 80%. To account for an expected response rate of approximately 70%, recruitment targeted 226 dentists.

### Settings and variables

This study employed a questionnaire-based survey. All participants who volunteered to enroll provided informed consent through a digital and anonymous form before completing the questionnaire. The questionnaire was created using Google Forms™ (Google, Mountain View, California, USA) and was structured into three parts:

A) Demographic aspects (age and city of work);
B) Professional aspects, including:

- Type of service (public/private);
- Presence of dental staff (assistants or technicians);
- Office accessibility (e.g. free access or receptionist/front desk control);
- Allowance of companions in the dental office;
- Working night shifts; and.
- Years of professional experience;

C) Exposure:

- Feeling intimidated in the workplace;
- History of being stalked;
- Exposure to psychological, physical, or sexual violence;
- Whether sought help; and.
- Need for psychological treatment.

#### Statistical analysis

Data were analyzed using descriptive statistics, presenting absolute (n) and relative (%) frequencies with a 95% confidence interval. Logistic regression models were employed to estimate odds ratios (ORs) for the likelihood of female dentists being exposed to violence, which was the primary outcome. The analysis considered subgroups of interest from the professional aspects (Part B) and exposure (Part C) sections of the questionnaire. Age was the only continuous variable and was included in the regression model to examine its relationship with the odds of experiencing violence. Statistical analyses were conducted at a 5% significance level using Stata™ software, version 18 (StataCorp LLC, College Station, Texas, USA).

## Results

A total of 165 female dentists responded to the survey, representing a 73% response rate. Most respondents reported experiencing some form of violence in their dental practice (*n* = 103, 62.4%). This exposure to violence created an intimidating environment for most dentists (*n* = 95, 75.8%). The highest rates of violence were observed among dentists working in the public sector (78.8%), with assistants (65.1%), with more than 10 years of experience (65.2%), and who had been stalked by patients or their companions (74.7%) (Table 1).

**Table 1:** Violence against female dentists related to professional aspects and exposure.

	**Total sample**			**Experienced violence**		
	**N**	**%**	**CI 95%**	**N**	**%**	**CI 95%**
**Have you ever experienced violence from patients or their companions in clinical practice?**						
No	62	37.6	30.5; 45.3			
Yes	103	62.4	54.7; 69.5			
**Which sector do you work in as a dentist?**						
Public	33	20.0	14.5; 26.9	26	78.8	61.6; 89.6
Private	111	67.3	59.7; 74.0	64	57.7	48.2; 66.6
Both	21	12.7	8.4; 18.8	13	61.9	40.1; 79.8
**Do you work with dental staff, such as assistants or technicians?**						
No	59	35.8	28.8; 43.4	34	57.6	44.7; 69.6
Yes	106	64.2	56.6; 71.2	69	65.1	55.5; 73.6
**Does your office allow patients to enter freely?**						
No	64	38.8	31.6; 46.5	41	64.1	51.6; 74.9
Yes	101	61.2	53.5; 68.4	62	61.4	51.5; 70.4
**Do you allow companions to accompany patients during dental appointments?**						
No	24	14.5	9.9; 20.8	16	66.7	46.0; 82.5
Yes	141	85.5	79.2; 90.1	87	61.7	53.4; 69.4
**How many years of experience do you have as a dentist?**						
0-9 years	96	58.2	50.5; 65.5	58	60.4	50.3; 69.7
10 years or more	69	41.8	34.5; 49.5	45	65.2	53.2; 75.5
**Have you ever experienced stalking by patients or their companions?**						
No	82	49.7	42.1; 57.3	41	50.0	39.3; 60.7
Yes	83	50.3	42.7; 57.9	62	74.7	64.2; 82.9
**Do you work night shifts?**						
No	90	54.5	46.8; 62.0	55	61.1	50.6; 70.7
Yes	75	45.5	38.0; 53.2	48	64.0	52.5; 74.1
**Have you ever felt intimidated by patients or their companions during your work?**						
No	37	22.4	16.7; 29.5	6	16.2	7.4; 31.8
Yes	128	77.6	70.5; 83.3	97	75.8	67.6; 82.5

N: absolute frequency; %: relative frequency; CI: confidence interval.

Statistically significant associations in this study indicated that female dentists who experienced intimidation or stalking by patients (or their companions) had higher odds of exposure to violence. Specifically, intimidation increased the likelihood of experiencing violence by 16 times compared to those who were not intimidated before (*p* < 0.001). Similarly, being stalked increased the likelihood of experiencing violence by three times (*p* = 0.001) (Table 2).

**Table 2:** Associations between the variables of interest and the outcome of being exposed to violence.

	**OR**	**CI 95%**	**p**
**Which sector do you work in as a dentist?**			0.076
Public	1.00		
Private	0.37	0.15; 0.92	
Both	0.44	0.13; 1.47	
**Do you work with dental staff, such as assistants or technicians?**			0.344
No	1.00		
Yes	1.37	0.71; 2.63	
**Does your office allow patients to enter freely?**			0.729
No	1.00		
Yes	0.89	0.47; 1.71	
**Do you allow companions to accompany patients during dental appointments?**			0.640
No	1.00		
Yes	0.81	0.32; 2.01	
**How many years of experience do you have as a dentist?**			0.529
0-9 years	1.00		
10 years or more	1.23	0.65; 2.34	
**Have you ever experienced stalking by patients or their companions?**			0.001
No	1.00		
Yes	2.95	1.53; 5.70	
**Do you work night shifts?**			0.703
No	1.00		
Yes	1.13	0.60; 2.13	
**Have you ever felt intimidated by patients or their companions during your work?**			< 0.001
No	1.00		
Yes	16.17	6.17; 42.36	

It is notable that nearly 72% of the overall sample did not seek help. However, among those who did, almost half (46.8%) required psychological treatment. A significant association was found between seeking help and the need for psychological treatment (*p* < 0.001). Women who sought help were 4.06 times more likely to require psychological treatment compared to those who did not seek help. A significant association was also observed regarding years of professional experience (*p* = 0.034). Dentists with 10 or more years of experience were 56% less likely to require psychological treatment following an incident of violence compared to those with 0–9 years of experience (Table 3).

**Table 3:** Association between the exposure to violence and the need for subsequent psychological treatment.

	**Total sample**			**Needed psychological treatment**					
	**N**	**%**	**CI 95%**	**N**	**%**	**CI 95%**	**OR**	**CI 95%**	**p**
**Needed help**									< 0.001
No	118	71.5	64.1; 77.9	21	17.8	11.9; 25.8	1.00		
Yes	47	28.5	22.1; 35.9	22	46.8	33.1; 61.0	4.06	1.93; 8.54	
**Years of experience**									0.034
0-9 years	96	58.2	50.5; 65.5	31	32.3	23.7; 42.3	1.00		
10 years or more	69	41.8	34.5; 49.5	12	17.4	10.1; 28.3	0.44	0.21; 0.94	

N: absolute frequency; %: relative frequency; CI: confidence interval; OR: odds ratio; p: statistical significance set at 5%.

**Fig. 1:**
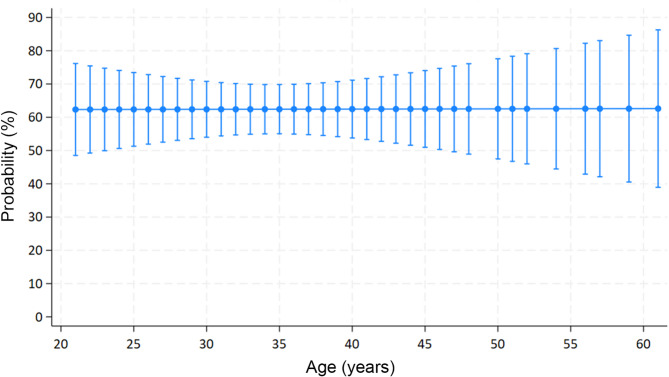
Probability of female dentists being exposed to violence in the clinical practice in Brazil according to their age. Legend: Stable probability around 62% was observed showing the exposure to violence can affect female dentists at any point in their career despite the age.

The analysis by age indicated that female dentists are at risk of exposure to violence throughout their careers, as the prevalence of violence exposure remained consistently around 62% across different age groups (Fig. 1).

## Discussion

Approximately 85% of the female dentists sampled in this study reported that they typically allow companions to be present during dental treatment. Although allowing companions was not significantly associated with exposure to violence, being stalked by patients or their companions was significantly associated. The term “stalking” is estimated to date back to the 15th century, originally used to describe stealthy hunting. Since the 1980 s, the term “stalking” has been used to describe obsessive harassers (https://www.etymonline.com/*).* A recent systematic review of mental health care professionals reported stalking prevalence rates between 10.2% and 50%.^19^ The present study found a stalking prevalence of 50.3% among female dentists. One possible explanation for this high rate is because, in the general population, females are disproportionately targeted for stalking compared to males^19^. Authors have reported that between 25% and 35% of stalking cases involving healthcare providers escalate to violence^20^. In the present study, nearly 75% of female dentists with a history of stalking by patients or their companions reported violence incidents. This alarming rate suggests that stalking may be a red flag, indicating high risk of subsequent violence against female dentists. This theory was supported by statistical analysis, which showed that stalking increases the likelihood of experiencing violence by up to three times.

Stalking or stalking behaviors are considered criminal offenses in several countries, including the United Kingdom, United States, France, Italy, Germany, Japan, and Brazil. In 2021, Brazil introduced the Federal Law No. 14,132, which defines stalking as: “*Persistently pursuing someone*,* by any means*,* threatening their physical or psychological integrity*,* restricting their mobility or*,* in any way*,* invading or disturbing their freedom or privacy*”. Even after the implementation of the law, Brazil recorded one case of stalking against women every 6 min and 48 s in 2023—an increase of 34.5% compared to the previous year. Healthcare providers who are victims of stalking often experience a negative impact on their confidence and professional competence^19^. To mitigate this problem, various strategies have been proposed. One modern solution designed to enhance the safety of victims, particularly women, is the FollowItApp™ (https://followitapp.org.uk/*).* This digital tool, developed in alignment with Scottish law, includes features such as geolocation, secure storage of photos and videos, fields for written documentation, and quick communication with the police. In addition to this tool, establishing a single and straightforward channel (such as a hotline with the Federal Council of Dentistry or the National Dental Union) would represent a significant step towards the protection of victims. It must be noted, however, that these strategies only represent potential applications that could be explored in future research but were not related to the objectives of the present study.

Regarding the increased likelihood of violence against female dentists, this study identified “feeling intimidated” in the dental office as the strongest predictive factor—raising the risk of exposure to violence by 16 times compared to those who did not report such feelings. While stalking may involve more subtle behavior, intimidation tends to be more overt. In this study, intimidation was analyzed as a potential early warning sign of future violence, a hypothesis that was statistically confirmed. However, in practice, intimidation can already be considered a form of psychological violence^21^. This type of violence can be verbal or non-verbal. Verbal intimidation includes threats, raised voices, and false accusations intended to damage the dentist’s reputation—often amplified through online platforms. Non-verbal intimidation may involve standing in a threatening posture, refusing to leave the dental office, or making aggressive gestures. In this context, (video) recording clinical consultations has emerged as a potential strategy to document patient behavior and provide evidence to support the dentist in cases of violence. This type of protection depends on the specific legislation of each country, including regulations on patient image rights and informed consent, and must uphold confidentiality^22^. However, simply informing the patient that the consultation is being recorded can already serve as a deterrent to harmful behavior. It is important to note that intimidation is often facilitated by opportunity and specific circumstances. For this reason, the present study also examined variables that, while not statistically significant, remain highly relevant to risk assessment. Working alone (without dental staff), operating without front desk control, and working night shifts are all conditions that may increase the risk of violence. Rather than suggesting that women avoid these situations—an approach that could disrupt their professional routines and financial stability—this study calls on public security agencies and dentistry-related organizations to advocate for female professionals and promote safer work environments. Approximately 28% of the women surveyed reported needing help after experiencing some form of violence in their dental practice. Psychological treatment was strongly associated with the type of assistance sought. This finding underscores the importance of previously suggested preventive strategies, such as the use of monitoring software and clinical video recording. However, given the persistently high rates of violence against women, immediate remediation strategies are also essential. One example would be the inclusion of regular psychological support sessions in the schedules of female dentists, particularly those working in public services, where patient volume is typically higher. This would provide a safe space for professionals to seek support and guidance.

Another point of concern emerged from the analysis based on years of experience, which showed a higher likelihood of requiring psychological treatment among female dentists with fewer years of practice. This finding may suggest that more experienced professionals tend to internalize their negative experiences with patients, whereas newer generations of female dentists are more likely to communicate and seek help. Sharing trauma with a trained mental health professional can be a crucial first step toward coping with these experiences and preventing the accumulation of stress and inherent physical manifestations.

This study has limitations that should be acknowledged. First, the use of self-reported data may introduce recall and reporting biases, as participants might underreport or overreport experiences of violence due to memory lapses or social desirability. Second, the sample was limited to female dentists practicing in the State of São Paulo, which may affect the generalizability of the findings to other regions or countries. More specifically, São Paulo may have different rates of violence compared to other areas, which could contribute to levels of perceived insecurity and workplace violence among participants that are region-specific. Consequently, the prevalence rates observed in this study might not fully represent the experiences of female dentists working in regions with different social and security contexts. Future research should consider broader geographic samples and incorporate objective measures to further validate these findings.

## Conclusion

Almost two-thirds of the female dentists surveyed in this study reported a history of violence involving patients or their companions. Stalking and intimidation were associated with higher odds of reported violence exposure, by approximately threefold and sixteenfold, respectively. Less experienced professionals were more likely to seek help, and psychological treatment was the most common form of assistance among those who did.

## Electronic supplementary material

Below is the link to the electronic supplementary material.

Supplementary Material 1

## Data Availability

The datasets used and/or analysed during the current study are available from the corresponding author on reasonable request.
